# Supplementary figures and images for: Parental age selection in C. elegans influences progeny stress resistance capacity
Source: bioRxiv. 2025 May 6:2025.04.30.651556. Preprint. [Version 1] doi: 10.1101/2025.04.30.651556 (PMC12247647; doi:10.1101/2025.04.30.651556)

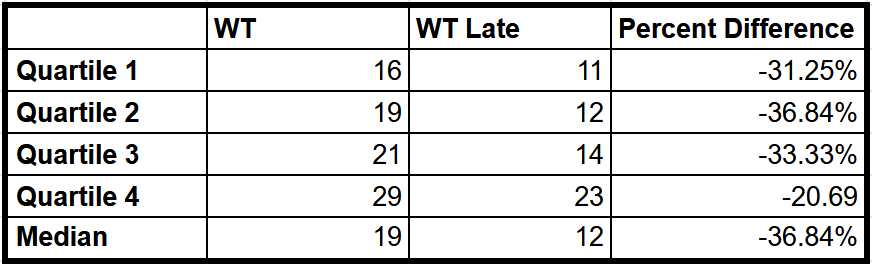

Supplement: Supplement 1 [file media-1.tif]
