# Supplementary material for: Parental age selection in C. elegans influences progeny stress resistance capacity
Source: bioRxiv. 2025 May 6:2025.04.30.651556. Preprint. [Version 1] doi: 10.1101/2025.04.30.651556 (PMC12247647; doi:10.1101/2025.04.30.651556)
Supplement: Supplement 3 [file NIHPP2025.04.30.651556v1-supplement-3.pdf]

bioRxiv preprint doi: https://doi.org/10.1101/2025.04.30.651556; this version posted May 6, 2025. The copyright holder for this preprint (which was not certified by peer review) is the author/funder, who has granted bioRxiv a license to display the preprint in perpetuity. It is made available under aCC-BY 4.0 International license.

SUPPLEMENTAL FIGURES

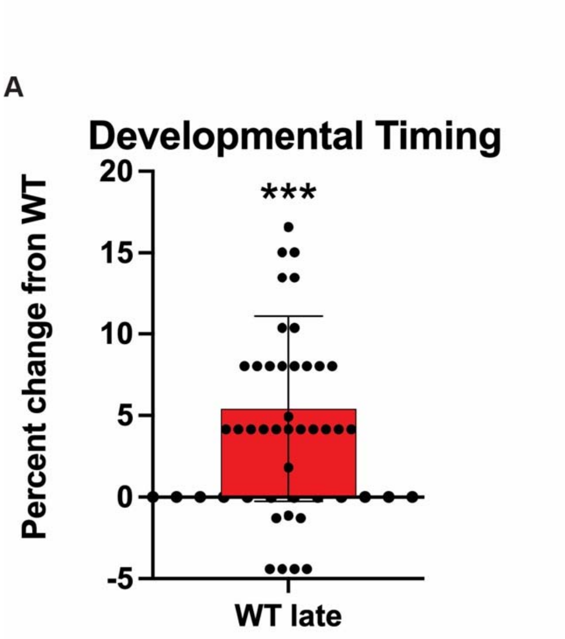

Figure S1: (**a**) WT Late worms have a slightly increased time till first egg lay compared to WT worms

bioRxiv preprint doi: https://doi.org/10.1101/2025.04.30.651556; this version posted May 6, 2025. The copyright holder for this preprint (which was not certified by peer review) is the author/funder, who has granted bioRxiv a license to display the preprint in perpetuity. It is made available under aCC-BY 4.0 International license.

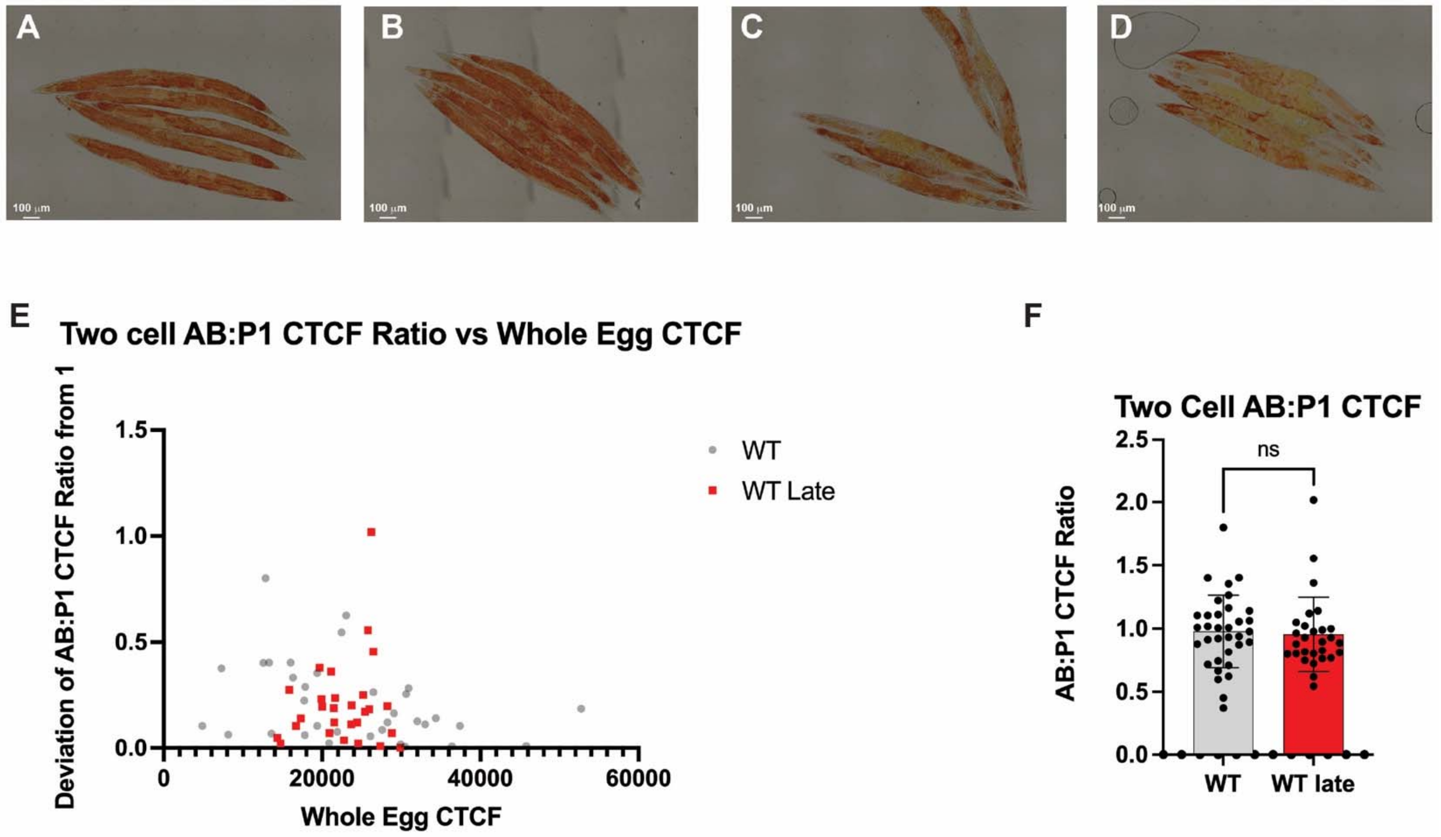

Figure S2: (**a**) WT non-asdf worms (**b**) WT Late non-asdf worms (**c**) WT Late intermediate Asdf worms (**d**) WT Late Asdf worms (**e**) WT Late worms have no significant change in the distribution of Nile Red stained lipid distribution between their AB and P_1 cells and the distribution is not dependent on the total lipid content of the egg (**f**) WT Late worms have no significant change in the distribution of Nile Red stained lipid distribution between their AB and P_1 cells

bioRxiv preprint doi: https://doi.org/10.1101/2025.04.30.651556; this version posted May 6, 2025. The copyright holder for this preprint (which was not certified by peer review) is the author/funder, who has granted bioRxiv a license to display the preprint in perpetuity. It is made available under aCC-BY 4.0 International license.

SUPPLEMENTARY TABLES

Table S1: Quartiles for WT and WT Late lifespan assay

Table S2: *gst-4::gfp* reporter RNAi of GWS hits results
